# The Question of HIV Vaccine: Why Is a Solution Not Yet Available?

**DOI:** 10.1155/2024/2147912

**Published:** 2024-04-08

**Authors:** Martina Libera, Valeria Caputo, Giulia Laterza, Louiza Moudoud, Alessio Soggiu, Luigi Bonizzi, Roberta A. Diotti

**Affiliations:** ^1^One Health Unit, Department of Biomedical, Surgical and Dental Sciences, School of Medicine, University of Milan, Via Pascal 36, 20133 Milan, Italy; ^2^Pomona Ricerca S.r.l, Via Assarotti 7, 10122 Turin, Italy; ^3^Department of Clinical and Community Sciences, School of Medicine, University of Milan, Via Celoria 22, 20133 Milan, Italy; ^4^SC Maxillo-Facial Surgery and Dentistry, Fondazione IRCCS Cà Granda Ospedale Maggiore Policlinico, Via Francesco Sforza 35, 20133 Milan, Italy

## Abstract

Ever since its discovery, human immunodeficiency virus type 1 (HIV-1) infection has remained a significant public health concern. The number of HIV-1 seropositive individuals currently stands at 40.1 million, yet definitive treatment for the virus is still unavailable on the market. Vaccination has proven to be a potent tool in combating infectious diseases, as evidenced by its success against other pathogens. However, despite ongoing efforts and research, the unique viral characteristics have prevented the development of an effective anti-HIV-1 vaccine. In this review, we aim to provide an historical overview of the various approaches attempted to create an effective anti-HIV-1 vaccine. Our objective is to explore the reasons why specific methods have failed to induce a protective immune response and to analyze the different modalities of immunogen presentation. This trial is registered with NCT05414786, NCT05471076, NCT04224701, and NCT01937455.

## 1. Introduction

The HIV-1 pandemic has persisted as one of the world's greatest health threats since the early 1980s, and after almost 40 years of intensive research, neither a resolutive cure nor a vaccine have been developed. Despite the important role that pre-exposure prophylaxis (PrEP) and antiretroviral therapy (ART) play, it should not distract the scientific community from developing a vaccine for HIV-1 eradication [1]. Developing countries have the highest incidence of infection, and about nine out of 10 sick people cannot afford these life-lasting medications [1, 2]. In addition, the urgent progression toward a HIV-1 vaccine is also due to some important pharmacological limitations such as the occurrence of drug-resistant viral variants, adverse effects, and interactions with different medications [3]. Since 1796, the traditional workflow of isolating and inactivating or attenuating the virus, followed by injection, was found to be extremely effective and, in the past, it was applied to treat HIV-1 infection with inconclusive results [4]. Due to this failure, many other immunogens and different vaccines have been proposed and studied, but up today an effective anti-HIV-1 vaccine is not yet available. However, it is important to consider that the advancement of vaccine, but also treatment, development is hindered by the high genetic variability of HIV-1. This peculiar characteristic of HIV-1, manifested as viral quasispecies, enables the virus to evade both immune responses and treatment pressures [5]. This high diversity is influenced by several factors, with the most significant being the high mutation rate [6] and retroviral recombination [7]. The rate of error-prone nature of reverse transcriptase in HIV-1 is approximately 1.4 × 10^−5^ errors per base pair per replication cycle [6]. This translates to approximately 0.1 mutations synthesized per genome. Alongside, mutations can also be introduced by the influence of mutagenic factors within host cells, such as APOBEC3G [8]. Nevertheless, the adaptive flexibility of HIV-1 cannot be solely explained by mutations. In fact, retroviral recombination effectively drives the evolutionary mechanism [7].

The pivotal role of retroviral recombination mechanisms is to efficiently mix mutations within a quasispecies, thereby leading to the evolution of fitter forms that contribute to drug resistance [9].

This heterogeneity has been classified into groups, subtypes, and recombinant forms of the virus. There are currently four major groups of HIV-1 (M, O, N, and P), with group M being the most prevalent and diverse [10, 11]. Within group M, there are nine subtypes and numerous circulating recombinant forms that result from recombination events between different subtypes [12]. The HIV-1 Env protein is certainly the protein most prone to genetic variations, as the immune response primarily targets this protein. Indeed, genetic diversity, which translate into antigenic variation, helps HIV-1 to evade immune detection. Typically, the onset of infection involves a relatively uniform viral population, characterized by less than 1% diversity in the *env* gene. As the asymptomatic phase progresses, this viral population undergoes diversification, peaking at up to 10% diversity in the *env* gene and increasing divergence. Subsequently, there is a stabilization or decrease in population diversity and divergence as the infection advances toward the AIDS phase [13]. Moreover, it was observed lengthening in the variable region V1V2 loop during the infection before declining in late-stage illness [14]. Notwithstanding, some other key factors contribute to the difficulty to develop effective vaccines. First off, the Env protein is composed of the outer envelope glycoprotein (gp120) and the transmembrane glycoprotein (gp41) and forms a trimer on the surface of the virus [15]. This heterotrimer is covered by approximately N-linked glycans, providing a shield that protects the underlying protein from immune recognition [16]. Moreover, HIV-1 displays around 14 trimers per virions, minimizing the immunogenicity of the particle [17]. Lastly, conformational flexibility of the Env protein during the attachment and fusion steps that help masking antibody epitopes [18].

In this scenario, it is simple to conclude that developing a vaccine capable of eliciting such a broad response is not easy.

In this review, we want to show the various immunogens studied, trying to explain the reasons why these strategies were unsuccessful according to the specificity of this virus. We will discuss from traditional to the most innovative vaccine strategies aimed at inducing a protective immune response.

## 2. Traditional Vaccinal Approaches

### 2.1. Inactivated and Attenuated Vaccines

The traditional vaccine approaches based on the inactivation or attenuation of the pathogen had been successfully used since 1796, and they were initially attempted also for HIV-1. Although the killed whole-virus vaccine strategy has been neglected due to risks associated with partial viral inactivation and other technical challenges, one of the first adopted strategies was based on formaldehyde-inactivated HIV-1 in mice models. However, it was unsuccessful especially due to a cytotoxic effect of induced sera, which compromised the efficacy evaluation *in vitro* [19, 20]. One of the first HIV-1 vaccines developed with a traditional approach was meant to be therapeutically administered to individuals who had already contracted HIV-1. Differently to prophylactic vaccines (useful to prevent the infection), the therapeutic approaches try to effectively eradicate the infection by modifying the immune response. The first therapeutic application of the inactivated virus, that was achieved through the chemical modification of gp120, was Remune, which phase III trial started already in 1997 [21]. Although initial results showed that combining this therapeutic vaccine with antiviral treatment led to an undetectable HIV-1 level, phase III trial was stopped because no difference between treated and untreated patients was observed [21, 22].

More recently, the safety and immunogenicity of a killed whole-HIV-1 vaccine (SAV001) were demonstrated in HIV-1 seropositive patients, underscoring the vital importance of preserving the natural structure of the envelope glycoproteins. This clinical trial has built upon a traditional killed whole-HIV-1 preparation, which underwent specific modification in the viral genome (Table 1) to address safety concerns and achieve the necessary viral quantity. Notably, these modifications included the removal of the *nef* gene from the HIV-1 genome and the replacement of the envelope glycoprotein signal peptide gene. Following these genetic changes, the viral inactivation process involved chemical treatment and gamma irradiation, aimed at retaining the authentic viral conformation [31]. This clinical trial demonstrated that a vaccine based on killed whole HIV was able to induce a neutralizing immune response, which marks a significant initial step in the development of new vaccines [32]. However, no information about additional studies was provided.

Attenuated strategies were considered for the constitution of an effective vaccine, but evidences on a naturally occurring attenuated *nef*-deleted HIV-1 strain demonstrated immunologic damage in patients [33]. Moreover, there is the possibility of the integration of the proviral DNA of live attenuated virus into the host chromosomal DNA, establishing a persistent infection [31].

### 2.2. Protein-Based Vaccines

Other traditional approaches include protein-based vaccines, which focus on the main viral proteins targeted by humoral immune response. As will be further discussed, the role and the importance of the humoral response in protecting from infection was extensively demonstrated by *in vivo* and *in vitro* experimental studies [34]. For a hypervariable virus like HIV-1, a successful vaccine must induce antibodies (Abs) able to interfere with a broad spectrum of HIV-1 strains [4].

In the context of HIV-1, the principal targets of the immune system are the Env proteins (gp160, gp120, and gp41) [35–37]. The functional Env is a trimeric structure on HIV-1 virion surface, consisting of three noncovalently associated gp120 and gp41 proteins. The precursor polypeptide, gp160, undergoes cleavage to produce two crucial proteins: gp120, responsible for binding to cellular receptors, and gp41, that mediates fusion peptide between viral envelope and cellular membrane through its fusion peptide [38]. Despite many studies conducted in this direction, several clinical trials demonstrated the poor immunogenicity of monomeric gp120 (e.g., AIDSVAX). This could be linked to the production and structural arrangements of recombinant proteins, a crucial aspect in developing a potent vaccine, as demonstrated by other vaccine preparations. Moreover, based on Env conformation, HIV-1 can be divided into four tiers that mirror the sensitivity to antibody-mediated neutralization. Among these tiers, viruses belonging to tier 1A display increased sensitivity to antibodies-mediated neutralization, whereas those belonging to tier 3 exhibit reduced sensitivity [39].

As a result, numerous investigations were undertaken to obtain soluble trimers structures that mimic the native conformation of HIV-1 Env protein. One of the most successful soluble stabilized Env trimer is known as BG505 SOSIP.664, which was achieved by introducing specific modification into the native sequence. Specifically, BG505 SOSIP.664 is a truncated form of gp160 Env obtained introducing a stop codon at position 664 to remove the C-terminal, the transmembrane domain, and membrane proximal external region (MPER) domain, necessary to guarantee the high solubility of the protein [40]. To establish a connection between gp120 and gp41, an intermolecular disulfide bond (SOS) was introduced by A501C and T605C mutations [41] while the substitution I559P (isoleucine to proline, IP) stabilized the prefusion form of the protein [42]. Moreover, the final formulation of this protein includes a REKR cleavage site to enhance proteolytic cleavage for the generation of gp120 and gp41 components. The additional T332N mutation (threonine to asparagine) was introduced to restore the binding affinity for broadly neutralizing antibodies (bNAbs) targeting N332 glycan, a specific portion of gp120. Initially accomplished to Env sequence from BG505 strain, this modification was subsequently extended to Env proteins derived from other strains as well [43]. Maintaining key attributes of the native Env protein, this recombinant protein served as the subject of both structural and immunological studies [42]. Due to its specific interaction with neutralizing antibodies (NAbs) as opposed to non-neutralizing antibodies, BG505 SOSIP.664 was employed in immunizing animal models, leading to the isolation of monoclonal NAbs with potential applications in both prophylactic and therapeutic contexts [44, 45].

In contrast, alternative approaches involved the design of a soluble Env protein that connected gp120 and gp41 by a flexible linker made of glycine and serine residues. Despite having a simpler structure than SOSIP, these proteins failed to primarily induce a neutralizing humoral immune response [46].

However, both preclinical and clinical studies demonstrated that Env struggles to elicit high Abs titers, with the anti-Env antibodies typically exhibiting shorter half-lives compared to those induced by other protein-based vaccines [47]. In addition, the humoral response triggered by Env immunogens neutralize only laboratory-adapted tier 1 viruses and/or the autologous virus harboring the Env variant used in the vaccine preparation, yet it does not extend to heterologous primary isolates [34]. This pattern has been validated through various comparative experiments involving both monomeric and native conformations of Env. While gp120 induces an adequate immune response, the resulting antibody tier is lower and declines faster than other immunogens, such as hemagglutinin [48, 49]. This impairment of the immune system could be attributed to the signaling triggered by the binding between gp120 and CD4. In fact, many studies reported that gp120 interferes with B cell maturation and proliferation *in vitro* [50]. Moreover, the poor immunogenicity of Env proteins arises from their unique properties.

Conserved regions on Env proteins are shielded by a coating of glycans, and vulnerable sites are transiently exposed. Furthermore, Env sequences can change without compromising function, hastening the viral evasion from immune system detection [51].

Given the suboptimal immunogenic features of Env proteins, several adjuvants have been explored. Specifically, studies have been carried out to assess both their impact on the immune system and on the maintenance of the structural integrity of immunogens. To date, research uniformly concludes that adjuvants enhance the immunogenicity of the Env proteins; however, distinct adjuvants are required for trimers and monomeric forms of Env proteins [51].

## 3. Vaccinal Approaches Based on the Structure of Anti-HIV Antibodies

Antibodies serve as a crucial immunological defense against infection, with NAbs playing a pivotal role in both the control of infections and for vaccine success. NAbs are naturally induced during HIV-1 infections and, when administered through passive transfer, they demonstrate the ability to protect animal models and affect ongoing HIV-1 infection in humans [34]. However, it is worth noting that about 10%–30% of HIV-1-infected individuals generate antibodies with broader activity, capable of neutralizing more than one strain [52], but only a small fraction of these subject is able to produce high levels of NAbs needed to block the infection [53]. This relatively low proportion of individuals with broadly neutralizing antibodies can be attributed to the unique characteristics of these antibodies. They undergo a complex maturation process, featuring somatic hypermutations (SHM) that can reach levels of up to 40%, and possess an elongated heavy chain complementary determining region 3 (CDRH3) [43]. Moreover, HIV has a glycan shell on its gp120, which serves as a protective barrier against recognition by the humoral system. The induction of antibodies against these glycans is exceptionally challenging because glycans are low immunogenic molecules. Furthermore, anti-HIV-1 glycan antibodies must specifically target the Env antigen while avoiding self-reactivity [54].

**Figure 1:**
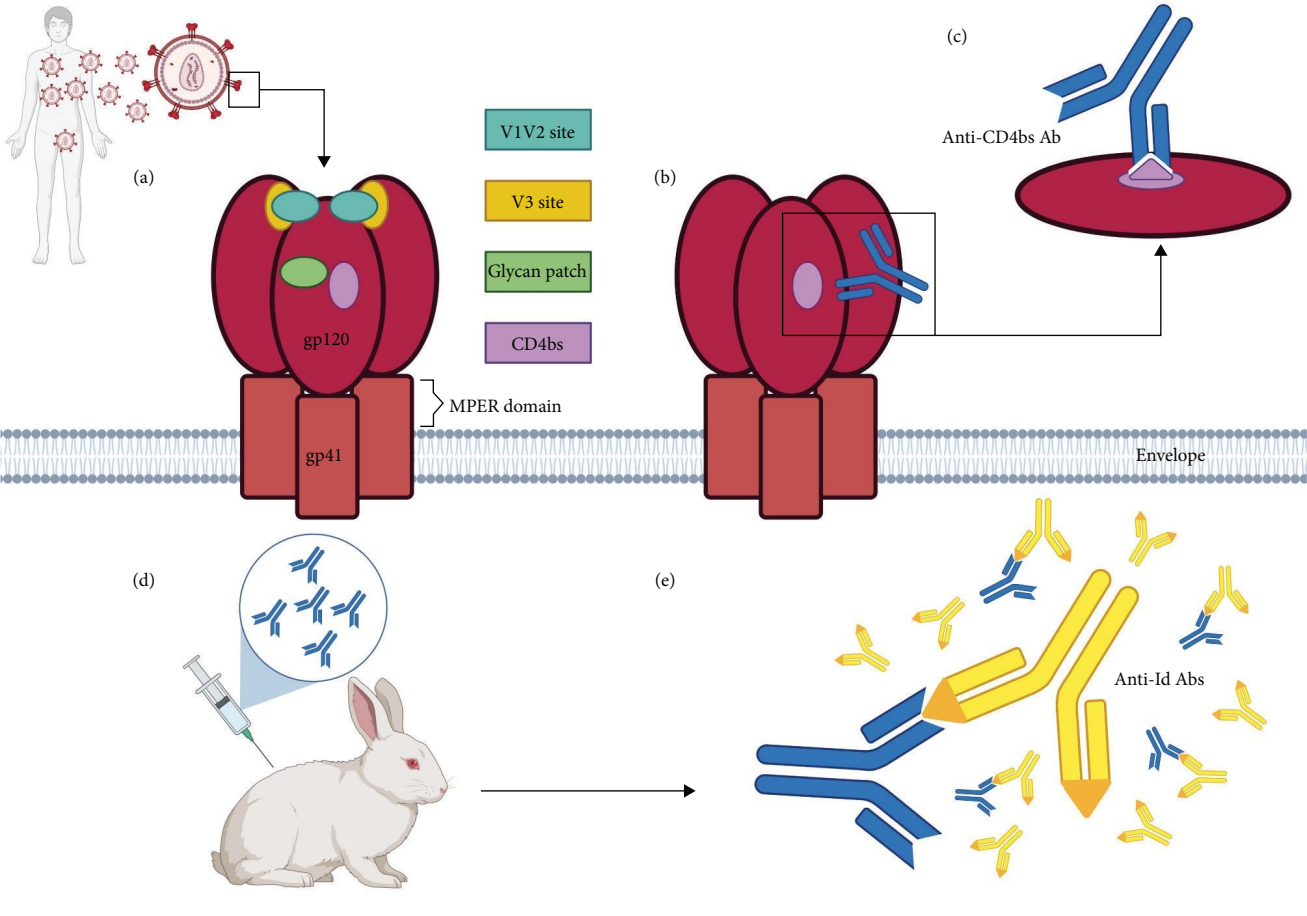
Schematic representation of functional Env and the generation of anti-idiotype antibodies. HIV Env is a trimer of heterodimers consisting of the surface glycoprotein gp120 and the transmembrane glycoprotein gp41 noncovalently associated. Located on gp120, there are major Abs binding sites reported in (a). The CD4bs is a target of the most potent bNAbs (b, c). Anti-CD4bs Abs (blue) can be used as immunogen for animal immunization (d) eliciting the production of anti-Id Abs (yellow) against recombinant and cellular human CD4 due to their capability to mimic CD4bs of the gp120 (e). Created with BioRender.com.

The quest for anti-HIV-1 NAbs began in the early 1990s, leading to the identification of first-generation NAbs, and subsequently, broader second-generation NAbs. This crucial breakthrough provided valuable insights into both the neutralizing humoral response and key sites of HIV-1. Through epitope mapping, researcher identified five major Abs binding sites: the CD4-binding site (CD4bs) [55–57], a viral glycan patch on gp120 [58], the MPER domain [59, 60], the hypervariable gp120 V3 loop [61], and V1/V2 gp120 loop (Figure 1) [59].

Consequently, vaccinal strategies based on immunogens that mimic these targets were developed, but the outcomes were disappointing, as they failed to elicit a broad or potent cross-clade humoral response [62]. These suboptimal results can be attributed to the unique characteristics of the NAbs described earlier [63–65]. Interestingly, anti-HIV-1 Abs that show absent to low levels of neutralization exhibit higher somatic mutations than nonspecific Abs cloned from the same B cell pool [34, 66]. However, it has been extensively observed that several mutations are necessary for achieving neutralizing activity [67–69], and that the level of SHM observed in HIV-1-specific Abs is higher compared to other chronic virus-specific Abs [70]. Hence, previous immunogens used to elicit bNAbs likely could not engage B cells expressing bNAb precursors and start their maturation [34].

Based on these observations, it was proposed to employ a series of vaccinations in a sequential manner. The purpose is to specifically target naïve B cell precursors and direct the immune response through multiple stages of development, mirroring the progression seen in a natural infection. To achieve this, a B cell-lineage vaccine design was developed.

### 3.1. B Cell-Lineage Vaccine Design

**Figure 2:**
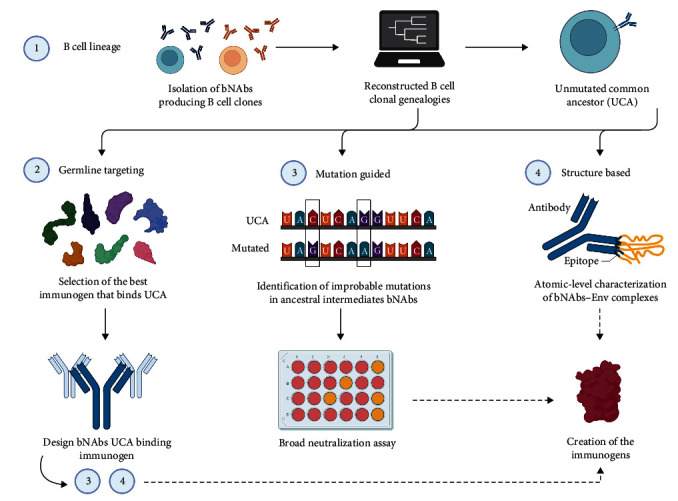
Schematic representation of different workflow strategies to produce new HIV-1 immunogens based on bNAbs strucure. Various antibody-based vaccination strategies have been used to develop new immunogens against HIV-1 infection. Although the techniques are numbered sequentially, there is no specific order in which they should be followed when creating novel immunogens. (1) B cell-lineage vaccine design. It involves the isolation of broad neutralizing antibodies (bNAbs) producing B cells and computationally reconstructing the whole B cell clonal lineages to replicate the molecular phylogenesis of the isolate bNAb, which will include all of its ancestral intermediates to the UCA. The ancestral intermediates and UCA act as molecular guides in immunogen development for different vaccine techniques such as germline-targeting, mutation-guided, and structure-guided immunogen design. (2) Germline targeting. Unfortunately, bNAbs UCAs cannot bind to most Env proteins, so germline targeting selects the best Env immunogen (by screening the Env variants library) that binds UCA and then uses it to construct a variety of high-affinity bNAbs precursors. The knowledge of the epitope structure can be improved using mutation-guided and structure-based immunogen design. (3) Mutation-guided immunogen design. It reflects the discovery of improbable broad neutralize mutations in ancestral intermediate bNAbs, which will be tested in an in vitro broad neutralization assay. Finally, mutated bNAbs will help to develop new immunogens. (4) Structure-based immunogen design. It consists of the atomic level characterization of the bNAbs-Env complex. It enables bioinformatic modeling of the Env immunogen around the Ab and its precursors to improve binding by introducing epitope alterations that result in the creation of the immunogen. Created with BioRender.com.

B cell-lineage vaccine strategy targets bNAbs-precursor B cells to guide the maturation of these antibodies through stimulation with Env-based immunogens. These immunogens are carefully chosen to bind each maturation intermediates [71, 72]. The initial step involves isolating a bNAb-producing B cell clone and sequencing its B cell receptors (BCRs) to identify related clonal members. The more clonal members identified, the easier it becomes to reconstruct the maturation history of the bNAb. However, because mature bNAbs undergo significant changes, especially the heavy (HC) and light chain (LC) CDR-3 regions, deducing the unmutated common ancestor (UCA) of the lineage can be challenging [53]. Computationally reconstructed B cell clonal genealogies play a fundamental role in studying Ab evolution and assisting in the design of HIV-1 vaccine. The design, based on UCA and the inferred ancestral intermediates, can be achieved by either germline-targeting, structure-based immunogen design, or mutation-guided immunogen design (Figure 2) [53].

Germline targeting entails selecting the best immunogen that binds the UCA. This priming step is critical, and the choice of the immunogen can be informed by longitudinal studies on HIV-1 positive patient defining the transmitted/founder (TF) virus or by the design the Env immunogen itself [73]. It has been observed that bNAb UCAs have limited binding affinity to most Env proteins, but may recognize the TF Env in some cases [74–76]. Alternatively, the immunogen can be engineered to enhance its binding avidity to UCA by screening Env variants library and gaining insights into the structure of the epitope. Both mutation-guided immunogen design and structure-based immunogen design contribute to this approach (Figure 2) [53]. An example of this strategy is the VRC01 class germline-based design. VRC01-class bNAbs stand out as the most potent and broad of all bNAbs discovered to date [77]. These antibodies specifically target the relatively conserved CD4-binding site epitope on the HIV-1 Env protein. They share a common HC V gene, VH1-202, which encodes a CDRH2 (complementary determining region 2, heavy chain) region that forms essential contacts with the CD4bs on Env. These HC are paired with a LC bearing a short five amino acid complementary determining region 3 (CDRL3) [68, 78, 79]. Importantly, VRC01-class precursors are relatively more common compared to other bNAbs precursors. In fact, they have been found in many HIV-1 patients at levels sufficient for germline targeting [80, 81]. Antibodies within this VRC01 class, such as VRC01 itself [78], 3BNC60 [68], 12A12 [68], N6 (broadest HIV-1 bNAb described to date [79]), have all been isolated from different patients. As expected, VRC01-class bNAbs showed a high level of SHM, including insertions and deletions, to achieve their remarkable activity and broad spectrum of neutralization. For the constitution of a vaccine capable of eliciting Abs like these, the best candidates developed so far were the engineered outer domain of gp120, eOD-GT8 [71], the glycan-deleted derivatives of clade C, 426c Env [82], and, most recently, two different versions of the near-native trimers BG505 SOSIP: BG505 SOSIP.v4.1-GT1 [83] and BG505 SOSIP.v4.1-GT1.2 [84]. The first three candidates have now been evaluated in human clinical trials (NCT05414786, NCT05471076, and NCT04224701, respectively). It was described that both eOD-GT8 and 426c Env immunogens primes VRC01-class precursors in knock-in (KI) mouse models [85, 86]; however, they engage different bNAbs precursors [87]. Despite their potential, these two immunogens are not able to select for insertion, and only eOD-GT8 is selected for single aa deletion in CDRL1 [88]. Instead, it has been proposed that Env trimer immunogens can favor improbable insertions [89]. The design of BG505 SOSIP.v4.1-germline-targeting trimer 1 is based on the BG505 SOSIP.664 and it engages both VRC01 and V2-apex germline-precursors. VRC01, PGV19, and NIH45-46 germlines were engaged with nanomolar affinity. However, this immunogen was not able to bind to other Abs of the VRC01-class [83]. A second modified version of BG505 SOSIP.v4.1-GT1, BG505 SOSIP.v4.1-GT1.2, has recently been developed to broaden VRC01-class precursor recognition. Maturation of the VRC01-class Abs has been reported, including rare multiresidue insertions that recapitulate those observed in VRC01-class bNAbs, as well as deletions and glycine substitutions [84].

Mutation-guided immunogen design aims to identify the improbable mutations in bNAbs that are critical for broad neutralization and convert this information into the design of immunogens capable of binding naïve or intermediated BCRs with the desired rare mutations [90].

Structure-based immunogen design is based on the characterization of bNAbs-Env complexes on an atomic level. This allows to model the Env immunogen around the Ab and its precursors to increase the binding by introducing epitope modifications. This approach is used to guide all stages of the vaccine design strategy [91]. An example of this approach based on V3 glycan-targeted bNAbs is PGT121. PGT121 is the best-characterized NAb exhibiting a high level of SHM which distinguishes it from other NAbs derived from the same precursor. It targets the region close to the V3 loop that is heavily glycosylated [92], and recognizes glycans at N301, N137, and N156, as opposite to other antibodies belonging to the same family that are highly dependent on N332 [93, 94]. Furthermore, while the precise identification of the actual UCAs of PGT121 class remains unknown, the presence of multiple members within the PGT121 lineage allows for the prediction of a PGT121 germline [93]. A structure-based approach was used to identify Env trimers variants with affinity for PGT121 germline-reverted precursors, resulting in an expansion of these precursors in PGT121 KI mice [95]. Moreover, sequential immunization with specifically Env designed immunogens induced NAbs maturation [96]. Following studies in animal models such as mice, rabbits, and rhesus macaques, showed that RC1 (engineered SOSIP Env trimers) and RC1-4fill (RC1 variant with glycans add to gp120 positions 230, 241, 289, and 344) induce clonal expansion of B cell expressing antibodies similar to inferred germline precursors of bNAbs targeting the V3-glycan patch [97]. The boosting experiment conducted on rhesus macaques, who were initially primed with RC1-4fill VLP, revealed that the immunization strategy utilizing the modified native-like Env trimer (SOSIP) resulted in the production of limited quantities of heterologous NAbs. Additionally, there were reports of off-target activity exhibited by these Abs [96].

### 3.2. Anti-Idiotype Antibodies

An additional vaccine approach against HIV-1 infection involves manipulating the immune network by utilizing anti-idiotype (anti-Id) Abs. Over the years, different pieces of evidence have suggested that idiotype–anti-idiotype interactions play a role in regulating immune responses for multiple infectious diseases. The underlying concept is that the idiotype region of any antibody is inherently antigenic: antibodies generated against the idiotype of the primary antibody molecule can structurally resemble the original antigen as they carry its “internal image” [98]. This set of internal image Abs can then be used as immunogens to induce protective immunity. Specifically, anti-Id Abs can serve as prophylactic vaccines themselves, or be employed to enhance existing humoral and cellular immune responses against selected epitopes (Figure 1) [99].

The use of anti-Id Abs as surrogate antigens has been shown to effectively stimulate a potential immune response that could confer protection against HIV-1. Murine monoclonal antibodies (mAbs) specific to p24, an abundant viral protein involved in HIV-1 assembly and maturation [100], were used to generate anti-Id Abs. Among these, TR009, an anti-Id Ab of 076 C, a murine mAb directed against p24, was found to recognize a common interspecies idiotype associated with anti-HIV-1 response. Immunizing rats with TR009 resulted in the induction of substantial antibody levels against recombinant p24 protein [99]. Another comparable approach was performed generating a series of overlapping anti-Id Abs targeting two anti-p24 murine mAbs, VIC5 and VIC6. Mice were immunized with a combination of the anti-Id Abs, which only slightly induced their reactivity to p24 antigen [101].

As previously mentioned, gp120 is a key target of anti-HIV-1 humoral responses and several vaccinal approaches based on this protein have been studied to induce a broad protective response. However, as already described above, none of the vaccines developed so far have reached this aim. To overcome this challenge, researchers have explored the concept of idiotypic mimicry employing antibodies directed against the idiotypes of antibodies able to bind CD4bs on gp120. A study provided a proof of concept for this strategy, in fact, antibodies directed against the idiotype of anti-gp120 immunoglobulins were used to mimic the conformation of CD4bs. Using these antibodies in the immunization of rabbits, antibodies capable of recognizing recombinant and cellular human CD4 were induced (Figure 1) [102].

The mAb 3C9, directed against the idiotype of human anti-CD4bs/gp120 Abs, was used to elicit bNAbs in naïve, non-HIV-1-infected monkeys [103]. However, the approach did not rely on anti-Id Ab mimicry, as 3C9 did not represent the internal image of the CD4 attachment site of gp120. Instead, 3C9 was found to define a marker on B cells producing anti-gp120 NAbs, thereby stimulating their synthesis. The study also introduced the concept that the ability of an anti-Id Ab to elicit antigen-specific immune responses depends on the presence of a common B-cell repertoire that recognizes both the antigen and anti-Id Abs in target animals [103].

An alternative strategy was based on the search of the anti-idiotype of the antibodies of long-term non-progressor (LTNP) patients, HIV-positive subjects able to maintain high CD4 and CD8 levels without specific therapy [104, 105]. Anti-CD4bs/gp120 IgG were purified from LTNP patients and used to immunize mice. This procedure allowed the identification of two anti-Id Abs, named P1 and P2, able to recognize the idiotype of both anti-CD4bs LTNP IgG and of one of the most broadly neutralizing human mAbs, IgG b12. Moreover, rabbit sera, obtained after immunization with P1, exhibited neutralizing activity of over 80% against HXB2 pseudovirus, and few of them were capable of neutralizing HIV-1-MN pseudovirus as well [104]

Studies on 1F7 idiotypes have revealed the significant role of anti-Id Abs as immune network regulators in the context of chronic infections. The 1F7 mAb is derived from mice immunized with immunoglobulin pool from several HIV-1-infected individuals [106]. Further investigations have demonstrated that the idiotype/clonotype 1F7 is commonly found in human monoclonal anti-gp120 and anti-p24 Abs, as well as in anti-HIV-1 human sera [107]. Various pieces of evidence support the biological relevance of the 1F7 idiotype in HIV-1 infection. A serological auto-anti-idiotypic humoral immune response to the 1F7 idiotype was observed using a specific peptide mimicking the idiotypic region [108, 109]. Anti-Id mAb 1F7 was shown to selectively inhibit anti-HIV-1 cytotoxic CD8^+^ T cells against uninfected CD4^+^ T cells, potentially contributing to the prevention of CD4^+^ depletion and disease progression in human HIV-1 infection [110]. Assessments of the 1F7 idiotype expression and evolution in patients' sera during acute and chronic HIV-1 infection have led to the conclusion that 1F7 expression is associated with a form of clonal dominance. This phenomenon appears to limit the development of NAbs against circulating infecting strains of HIV-1 [111]. bNAbs against HIV-1 have been found to express the 1F7 idiotype, suggesting that the same idiotypic axis, which underlies clonal dominance and original antigenic sin, also facilitates antibody evolution toward highly mutated progeny capable of neutralizing diverse strains of HIV-1 [111]. Understanding how to selectively exploit this axis to prime for the development of bNAbs prior to viral exposure is considered a promising strategy for the development of a more effective HIV-1 vaccination [111].

As previously mentioned, engaging naïve B cells capable of generating bNAbs is considered essential for the development of a successful HIV-1 vaccine. However, the approach of germline targeting using modified Env immunogens has faced limitations, particularly in terms of activating off-target immune responses [112]. Recent studies have shown that anti-Id Abs could recognize and activate bNAbs precursor B cells *in vivo*, making them a viable alternative or complement to Env immunogens. Anti-Id Abs specific to the inferred germline version of HIV-1 bNAb b12 (iglb12) were used to identify and analyze naïve B cells expressing iglb12-like BCRs. Immunization of transgenic mice with one of these anti-Id Abs resulted in the proliferation of transgenic murine B cells expressing the iglb12 heavy chain *in vivo*. Some instances of deletion and anergy were reported within this B cell population [113].

In an alternative approach, the anti-Id Ab iv8 was used to selectively expand a specific population of B cells, giving them a competitive advantage. This anti-Id Ab exhibited high affinity for VRC01-class precursor BCRs [112]. iv8 induced the expansion and maturation of B cells expressing VRC01-class Abs *in vivo*, resulting in serological responses targeting the CD4bs on the Env without activating B cell clones that produce off-target responses to Env [112].

An improved targeting strategy was implemented to optimize VRC01 precursors recognition. Initially, new anti-Id mAbs that bind with high-affinity multiple VRC01 precursors were developed. However, subsequent analysis revealed that these mAbs selectively recognize BCRs with either VRC01-class LCs featuring a 5-aa CDRL3 or with heavy chains derived from VH1-2^*∗*^02, but not BCRs possessing both characteristics [114]. The engagement and activation of B cells expressing VRC01 precursor-like BCRs were obtained through the engineering of a bispecific anti-idiotypic molecules derived from the anti-Id mAb iv9, that preferentially recognizes the VH1-2^*∗*^02 heavy chain, and the anti-Id mAb iv4 which tends to recognize LCs with 5-aa CDRL3 [114]. This bispecific anti-Id mAb iv4/iv9 activates iGL-VRC01 B cells *in vitro*, and in a murine adoptive transfer model, it displays superior engagement and expansion of VRC01 precursor B cells compared to iv4 or iv9 alone as Fab fragments [114].

Overall, these findings highlight the significance of the anti-Id mAbs as valuable immunogens for developing HIV-1, particularly for activating specific B cell lineages capable of producing protective antibodies.

## 4. *In Silico* Approaches for the T Lymphocytes Induction

Considering the continuous and constant improvement involving bioinformatic tools in these years, *in silico* analysis has been widely employed to study and develop immunogens. The use of these tools has previously been discussed in the context of studying immunogens based on the anti-HIV antibodies. Moreover, several studies have highlighted the importance of eliciting a T-cell response during HIV infection. Consequently, many vaccine approaches have been specifically directed toward achieving this objective.

One of the first strategies for the identification of cross-subtype HIV-1 immunogens was based on the individuation of conserved and promiscuous class II HLA-restricted T-cell epitopes from an HIV-1 Env sequences database. Following this, a set of “immunogenic consensus” was formulated, with some being synthesized and employed in *in vitro* systems, showing their immunogenicity. Consequently, the best candidates, most immunogenic and highly conserved, across a great number of HIV strains molecules were included in a vaccine preparation (the GAIA vaccine). Unfortunately, preclinical studies have not been conducted so far [115–117].

Another approach was used for the design of an mRNA vaccine based on epitopes of HIV-1 recognized by B- and T-cells. In this study, five HIV surface proteins were identified from the literature and, through bioinformatic tools, the most immunogenic epitopes able to activate humoral and cellular immune responses were found. Moreover, through *in silico* analysis, it was possible to study the complete vaccine preparation, and the immunological simulation revealed its capacity to elicit both innate and adaptive immune response. Using specific bioinformatic tools, the vaccine was also demonstrated to be able to establish memory cells upon exposure and to determine the production of chemokines responsible for the increasing of B cell and humoral responses [118].

Another bioinformatic study was based on the identification of specific anchor residue motifs in the primary amino acid sequence of the target pathogen involved in the binding to classes I and II HLA. The general idea for the vaccine constitution is the identification of conserved regions of pathogens and on the affinity prediction between prospective peptides and HLA molecules. Moreover, considering the high variability of HLA polymorphisms, peptides that could bind multiple HLA type were further studied to encompass the global population. When applied to HIV sequences present in the Los Alamos database for cytotoxic T lymphocyte (HTL) epitopes, the overall population coverage was about 98% using 17 epitopes. Epitopes immunogenicity was confirmed *in vivo* using HLA transgenic mice, and *in vitro* using PBMC-derived sera samples from HIV-1 exposed individuals [119]. Moreover, this strategy was improved by the application of a specific algorithm called TEPITOPE. It allowed the identification of a set of 18 HLA-DR binding CD4 T-cell epitopes derived from the whole clade B HIV-1 consensus protein-coding genome. All the selected epitopes were recognized by PBMC from HIV-1 infected patients [120]. These epitopes were used for the development of a DNA vaccine called HIVBr18, which elicit broad, polyfunctional, and long-living CD4^+^ T cell responses in BALB/c and HLA class II transgenic mice [121, 122]. Similar results were observed in rhesus macaques, where robust and predominantly polyfunctional CD4^+^ T cell responses were observed in all animals for 13 out of the 18 epitopes [123]. Overall, these studies establish the proof of concept that vaccines based on promiscuous and multiple T cell have the potential to induce immune responses with relevant breadth and depth. This represents an additional tool for the development of clinical studies.

## 5. Alternative Vaccination Strategies Based on Vectors

For the constitution of an effective vaccine, the selection of an appropriate immunogen to stimulate a strong, durable, and neutralizing immune response is undoubtedly a pivotal consideration. Nonetheless, there are additional crucial factors to consider, such as how diverse immunogens are presented to the immune system. Presently, vaccines have the potential to be created exclusively from the genetic materials responsible for encoding the desired immunogens. This innovative approach exploits the utilization of vectors, which encompass modified bacteria, viruses, plasmid DNA, RNA, or sequences from pathogens, serving as vehicles to deliver the target antigen [124].

In this section, we will focus on viral vectors based on DNA or RNA viruses, which found substantial application in clinical trials for developing vaccines targeting HIV-1. These vector platforms have emerged as the predominant choice in this regard (Table 2).

### 5.1. DNA Viruses

#### 5.1.1. Adenovirus Vectors

Adenoviral vectors (AdV) are probably the most known and tested platform used to carry an exogenous sequence encoding for an immunogen for vaccinal purposes and gene therapy [139]. To avoid potential risks related to recombination, which may enhance its pathogenicity, replication-defective (RD) human adenovirus serotype 5 (HAd5)-based vectors have been widely used in various vaccine trials [140]. The first proof of concept human clinical trials for HIV-1 prevention using HAd5V were carried out through the STEP trial (HVNT 502). This trial took place in geographical areas where clade B stands as the predominant subtype. Recently, a related study called Phambili trial (HVNT 503) was conducted in South Africa, where clade C is the prevailing subtype. Both trials assessed the efficacy of a vaccine containing three HAd5V able to express three HIV-1 proteins clade B (Gag, Pol, and Nef) (Table 1). Unfortunately, they were both stopped due to ineffectiveness: HIV acquisition appeared to be higher among patients with a preexisting anti-HAd5V antibody titer [121]. Despite the absence of definitive evidence, this phenomenon could be explained by the fact that vaccine can stimulate preexisting anti-HAd5V-specific CD4 T cells, thereby increasing the pool of cells permissible to HIV infection [121].

Given that approximately 90% of individuals possess existing immunity against HAd5 [139, 140] alternative AdVs utilizing fewer common serotypes, such as HAd26 and HAd35 have been formulated [127, 130]. After the aforementioned unsatisfactory results, these AdVs have mostly been associated with the advancement and evaluation of mosaic vaccines.

This last approach involves the development of a multivalent HIV vaccine utilizing a HAd26V-based (or Modified Vaccinia Ankara (MVA) based) vector. This vector encodes mosaic immunogens that have been computationally designed to trigger a neutralizing response against several HIV isolates [141]. Briefly, fragments from specific HIV-1 protein regions were designed using mathematical models, then combined to amplify the coverage of epitopes that T and B cells recognize. Moreover, it was demonstrated that a boost in the HAd26V-based immunization protocol with adjuvanted recombinant Env protein led to an increase in the neutralizing response in rhesus monkeys [142]. The APPROACH study was the first clinical trial aimed at evaluating the immunogenicity of a mosaic vaccine regime in order to prevent HIV-1 [143]. This pioneering trial not only verified the safety and tolerability of these formulations but also demonstrated the capacity to elicit antibodies directed specifically against the same HIV clade featured in the immunization. Nevertheless, the neutralizing activity was discernible solely against tier 1 HIV-1 isolates [144]. Starting from these important data, subsequent clinical trials were conducted to enhance the immunogenicity of these formulations, employing diverse combinations of immunization protocols [145] Regrettably, the MOSAICO phase III trial (HPX3002/HVTN706), conducted among transgender individuals and men who have sex with men, had to be prematurely halted due to its failure in preventing HIV infection.

This lack of efficacy was further corroborated by the phase IIb trial IMBOKODO (HPX2008/HVTN 705) that followed the same regimen and was conducted among young Sub-Saharan women [146, 147].

From this perspective, a distinct adenovirus derived from chimpanzees, specifically the Y25 strain, is currently under examination for its potential in vaccine therapy. One key advantage is its lack of connection to preexisting immunity in humans [148]. This vector, termed ChAdOx1, recently gained attention as a potential platform for COVID-19 vaccine development. Ewer et al. [149] demonstrated the elicitation of NAbs, with subsequent enhancement following booster doses. Outcomes are on the horizon for the development of an HIV-1 vaccine. ChAdOx1 has recently been within the context of the mosaic vaccine strategy HIV-1 prevention, as evidenced by studies such as HIV-CORE 0052 and HIV-CORE 006 (Table 3).

#### 5.1.2. Adeno-Associated Viral Vectors

Adeno-associated viral vectors (AAVs) have emerged as the preferred choice for both gene therapy and vaccine development due to their remarkable safety profile, minimal immunogenic characteristics, broad tropism (including postmitotic cells), and crucial ability to induce sustained Abs expression with a single administration owing to their episomal structure [140, 153]. AAVs have demonstrated lower immunogenicity than other viral vector platforms, although they retain the potential to elicit a dose-dependent immune response [154]. Despite the evident benefits outweigh the drawbacks, there is only one documented proof of concept clinical trial (NCT01937455) involving human subjects that used AAV for HIV-1 prevention in healthy volunteers [129]. This trial, which reached completion, involved the testing of different dosages of a recombinant AAV (rAAV) encoding for PG9, a human anti-HIV-1 broad neutralizing antibody (rAAV1-PG9DP) aiming to elicit a sustained expression of a specific immune response to prevent HIV infection. The vaccine proved to be safe, with PG9 detected in the sera of only four subjects, albeit at a low level [129].

Whether intended for gene therapy or as a platform for vaccines, the consensus among scientists is that more comprehensive research is imperative, employing a multidisciplinary approach to understand the innate immune response triggered by these vectors. Furthermore, an assessment of potential acute or chronic toxicity remains a necessary step [154].

#### 5.1.3. Pox Virus Vectors

MVA is a promising type of nonreplicant poxvirus developed as a vector vaccine in 1975 [1]. The viral distinctive ability to replicate within the host cytoplasm, coupled with its robust safety profile and broad tropism, makes the vaccinia virus an excellent foundation for vector-based vaccines. Approximately 15 years after MVA discovery, other nonreplicating vectors progressed, notably the highly attenuated vaccinia virus (NYVAC), and a canarypox virus (ALVAC) [1]. The latter is currently the most frequently used vector in HIV-1 clinical trials, along with HAd26 and MVA [1]. The most successful HIV-1 clinical trial, RV144 (or Thai trial), used the ALVAC vector expressing gp120 (subtype E) linking to gp41 (subtype B), gag and protease (subtype B)- for priming, followed by a booster administrating recombinant gp120 clade B protein/Alum (AIDSVAX® B/E) [155], resulting in a 31.4% efficacy rate [156]. Subsequent clinical trials were carried out in South Africa where HVTN 097 replicated the RV144 regimen and showcased comparable results; HVTN 100 and HVTN 702 introduced modifications related to the HIV-1 subtype selected for expressing immunogens and adjuvant employed. Notably, serotype C gp120 and MF59 were adopted in these trials. The decision to use serotype C was based on epidemiological study in South Africa, representing the first attempt at a regional-based improvement of the vaccine. Despite observing elevated levels of both humoral and cellular immune response in HVTN 100 (albeit distinct from those studied in RV144), the HVTN 702 trial, which sought to assess the effectiveness of this version of the vaccine, was prematurely terminated due to inconclusive results [135, 136, 157].

It becomes imperative to consider various factors such as the use of different adjuvants, the genetic diversity of Env gp120 genes, and the difference in HIV-1 incidence between South Africa and Thailand when embarking on the development of a preventive HIV-1 vaccine. This holds true even when employing the same type of vector and recombinant protein [143].

Being the sole successful HIV-1 clinical trial in preventing infection acquisition up to now, RV144 prompted two other studies (RV305 [131] and RV306 [134]) to explore the potential of post-trial immunizations for sustaining protective effects. RV305 and RV306 aimed to administer additional doses of ALVAC and AIDSVAX® B/E, either individually or in combination, 6–8 years or 12–15–18 months after the conclusion of RV144 trial, respectively [131, 134]. In contrast to the protocol regimen used in RV305, RV306 demonstrated that a booster can enhance the immune response within a year of RV144 vaccination regimen. Conversely, HVTN 702, mentioned earlier, yielded contrasting outcomes compared to RV306. Despite showing a 12-month booster, it failed to produce a statistically significant protective effect. Consequently, it is still uncertain whether the amplified responses observed in RV306 will translate into virus protection [158].

### 5.2. RNA Viruses

#### 5.2.1. mRNA Vector

The RNA technique is a recent type of genetic vaccine that holds the distinctive ability to deliver protein-coding sequence as messenger RNA (mRNA) [159]. The advent of the COVID-19 pandemic provided a global platform for evaluating and acknowledging this novel vaccine category, which is encapsulated in lipid nanoparticles to protect the molecule from degradation [159, 160]. mRNA as a vector offers greater efficiency compared to conventional vaccination strategies, largely due to its nonintegrating properties, its ease manipulation, and its extensive adaptability in generating various immunogens related to the pathogen under investigation [161]. Moreover, immunogens created from inactivated virus can be different from their native conformation, whereas subunit vaccines produced by nonhuman cell lines could introduce post-translational modifications that result in antigens bearing altered conformations [153]. Recently, the in-progress HVNT 302 clinical trial, situated in the United States, is evaluating three novel mRNA-based HIV-1 vaccines whose findings are expected by the end of 2023. The vaccine regimens use three versions of the stabilized native-like trimer [95]: the BG505 MD39.9 mRNA, the BG505 MD39.9 gp151 mRNA, and the BG505 MD39.3 gp151 CD4KO mRNA. Through these vaccines, the aim is to assess their safety and ability to elicit autologous NAbs.

In addition to previous applications, mRNA is also raising interest for its potential use in therapeutic clinical trials. In general, therapeutic vaccines have the unique characteristic to transform host immunity of chronic disease patients by either enhancing the T cell-mediated immune response or by stimulating B cells capable of producing specific Abs improving the humoral immunity [162, 163]. The reconsideration of these vaccines has lately been prompted by the failure of all HIV-1 prophylactic vaccine clinical trials, the rise of chronic diseases, and the development of drug resistances [162]. Although these vaccines could provide long-term immune memory to a large number of HIV-infected people, the virus exhibits few qualities that make it a challenging target for therapy success. Indeed, HIV-1 latency, Env proteins wide variability, viral genome diversity, and its rapid evolution make the progress of a therapeutic vaccine ambitious [162, 164]. Despite the difficulties, various therapeutic vaccine trials have been performed and few recent promising vaccines have completed at least phase I. Among them, the first-in-human dose-escalating clinical trial used naked mRNA targeting dendritic cells. It employed a novel HIV-1 immunogen (HTI) that encodes essential target epitopes found on Gag, Pol, Vif, and Nef (Table 1). The trial was performed in HIV-1 infected adults undergoing combined ART (cART) treatment (phase I iHIVARNA [137]). Results indicated that only the group receiving the highest dosage exhibited any effect on their T-cell response. All the other groups showed no observable changes. Given the proof of its safety, the same vaccine approach was replicated in a subsequent phase II trial, named iHIVARNA, which involved HIV-1 patients receiving cART, with the intention of discontinuing medication upon completion of the regimen [138]. However, the iHIVARNA product encountered an error in the RNA sequence. Unintentionally, a second start codon ahead of the HTI immunogen coding sequence was present, forcing the study to end earlier than expected. The consequences of this mistake would probably affect the expression of HTI protein, though the extent is still unknown [138].

Even if not in-depth discussed in this review, it is crucial to mention additional promising therapeutic vaccines that use different techniques than mRNA, such as peptide-based vaccines (VAC-3S, IPROTECT1, Vacc-4x) and DNA-based vaccines (VRC 101 study, DermaVir, BCN 01) [165]. Regardless of strategy, the ultimate objective of all therapeutic clinical trials is to induce CD8^+^ T cell-mediated responses, with particular attention to memory responses required for guaranteeing long-lasting immunity [165].

## 6. Conclusions

Vaccines hold the potential to elicit a protective response that ideally should be enduring. In the context of HIV-1, achieving this goal has remained elusive due to the peculiar biological characteristics of the virus that entailed the lack of success in numerous clinical trials conducted over the years. Nevertheless, the various experimental vaccination strategies, employing different formulations and approaches, have provided invaluable insight into the virus and its ability to trigger a humoral immune response. These insights are pivotal for the creation of vaccines aimed at significantly reducing the prevalence of HIV-1 infections.

As explored in this review, while traditional vaccination methods did not yield the desired outcomes, they did provide a pathway to comprehend the fundamental traits of HIV-1 Env protein and grasp its vulnerabilities. This understanding subsequently paved the way for the development of the most promising HIV-1 trial (RV144), which achieved a 31.4% efficacy rate. Despite its modest success rate, this trial marks a pivotal juncture in the pursuit of an effective vaccine for HIV-1, underscoring the importance of mirroring the evolutionary trajectory of the viral infection observed *in vivo*.

This notion was further explored in investigative studies that focused on targeting the naïve precursors and direct the humoral immune response through multiple maturation phases, mirroring the progression seen in natural infection. The identification of monoclonal neutralizing antibodies and anti-idiotypes played a pivotal role in shaping the development of this strategy. The advancements in antibody-to-vaccine and the combination of *in silico* and *in vitro* approaches are promising; however, numerous facets of this strategy are yet to be conclusively defined.

Vaccines designed to induce broadly neutralizing antibodies must trigger all different components of the immune response, as the CD4^+^ TFH cell response stimulates high levels of highly mutated bNAbs.

Numerous studies have examined the activation of T lymphocytes as a means of controlling and preventing infection, a perspective underscored by elite controller HIV-1-infected patients and diverse animal models. Nevertheless, clinical trials that aimed to induce T-cell responses experience setbacks, failing both in infection prevention and viral load management [166, 167]. Other investigations have explored the role of the innate immune system in constraining HIV-1, with a recent instance involving the examination of Toll-like receptor (TRL) agonists in conjunction with neutralizing antibodies for infection treatment or vaccination formulation [168, 169]. However, conclusive outcomes remain pending.

## 7. Future Recommendation

Although many strategies have been designed and tested, an effective anti-HIV-1 vaccine is still not available. These failures reflect the complexity of the virus itself and, even more, the interaction with its host. Vaccine development must take into account host-related factors that affect immune responses to HIV-1 vaccination and vaccine efficacy. Such factors encompass immunogenetics, body mass index, and other demographic variables. Additionally, the choice of vector and adjuvant within the vaccination protocol significantly impacts the potential immune response of recipients [157].

Despite the persistent effort to formulate an effective anti-HIV-1 strategy, the desired objective has yet to be attained. This underscores the presence of unidentified elements, but new insights from immunological studies is undoubtedly drawing us closer to that goal.

## Figures and Tables

**Table 1:** HIV-1 main genes and protein functions encoded.

Class	Gene	Polyprotein	Protein(s)	Function(s)	References
Viral structural proteins	*gag*	Gag	Matrix (MA, p17) Capsid (CA, p24) Nucleocapsid (NC, p7)	Encodes a polyprotein that is processed to form the structural proteins placed inside the envelope	[23, 24]
	*pol*	Pol	Protease (PR, p12) Reverse transcriptase (RT, p66) and RNaseH (p51) Integrase (IN, p32)	Proteolytic cut of Gag/Pol proteins. When in the cytoplasm, RT transcribes viral RNA into a complementary copy of DNA, resulting in the formation of dsDNA. RNaseH degrades viral RNA. In the nucleus, the integration of HIV dsDNA occurs into the host cellular genome by IN (formation of the provirus)	[24, 25]
	*env*	PrGp (gp160)	Surface protein (SU, gp120) Transmembrane protein (TM, gp41)	It fuses the viral and cell membranes after binding to the cellular receptor; gp160 undergoes a series of conformational changes, and it is cleaved in gp120 and gp41	[24, 26]

Essential regulatory elements	*tat*	—	Tat	Transactivator; activates transcription of viral DNA and cellular genes	[27]
	*rev*	—	Rev	Gene transcription regulator of virion proteins. Rev facilitates viral unspliced mRNAs transport from the nucleus to the cytoplasm for their translation	[25]

Accessory regulatory proteins	*nef*	—	Negative regulating factor	Nef is a protein that affects HIV replication, viral particle infectivity, CD4 downregulation and apoptosis	[24]
	*vif*	—	Viral infectivity factor	Vif regulates virus infectivity through the degradation of human APOBEC3G (cellular cytidine deaminase) protein, which provide innate immunity against various pathogens	[28]
	*vpr*	—	Viral protein R	Vpr controls the import of the HIV-1 dsDNA preintegration complex into the nucleus, the activation and apoptosis of infected cells, virus transcription, and cell cycle arrest	[29]
	*vpu*	—	Viral protein U	Vpu increases the release of viral particles from human cells and contributes to the overall downregulation of CD4s expression throughout HIV-1 infection by inducing their degradation	[30]

**Table 2:** Summary of the vectors mentioned in the “Alternative Vaccination Strategies Based on Vectors” section.

Molecule	Vector type	Study ID	Clinical trials identifier	Year started	Immunogen/description	Country	Population enrolled	Note		References
DNA viruses	Adenovirus	HVNT 502	—	2004	Human adenovirus-based vector type 5/HIV-1 proteins clade B (Gag, Pol, Nef) MRKAd5 HIV-1gag/pol/nef trivalent vaccine	US, The Caribbean, Australia	Homosexual men and high-risk heterosexual men and women		Clinical trial stopped	[125]
		HVNT 503	NCT00413725	2007	Human adenovirus-based vector type 5/HIV-1 proteins clade B (Gag, Pol, Nef) MRKAd5 HIV-1gag/pol/nef trivalent vaccine	South Africa	Healthy adults of both sexes		Clinical trial stopped	[126]
		IPCAVD001	NCT00618605	2008	Assess safety and immunogenicity of the recombinant adenovirus serotype 26 (Ad26.ENVA.01) which contains a HIV-1 Clade A *Env* gene encoding a modified envelope gp140 protein	US	Healthy adults of both sexes		—	[127]
		HVNT 505	NCT00865566	2009	Recombinant adenoviral serotype 5 (rAd5) vector vaccine encoding HIV-1 clade B Gag/Pol and HIV-1 clade A, B, C Nef/Env	US	Healthy, circumcised men, and male-to-female transgender persons who have sex with men		—	[128]
	Adeno-associated virus	IAVI A003	NCT01937455	2014	Recombinant AAV vector coding for PG9 antibody which is a human monoclonal IgG1 antibody that reacts with the V1V2 loop of the HIV-1 envelope gp120 protein and was derived from a patient with clade A HIV infection rAAV1-PG9DP	UK	Healthy adult males		—	[129]

DNA viruses	Pox virus	RV144	NCT00223080	2003	Prime: recombinant canarypox vector vaccine (ALVAC-HIV (vCP1521)) expressing subtype E HIV-1 gp120 (strain 92TH023) linked to the transmembrane anchoring portion of gp41 (strain LAI), and HIV-1 gag and protease (LAI strain) Boost: AIDSVAX® B/E, a highly purified mixture of gp120 proteins	Thailand	Healthy adults of both sexes		P_AE/B_/alum	[130]
		RV305	NCT01435135	2012	6–8 years late boost since RV144 vaccination: ALVAC-HIV + AIDSVAX® B/E, AIDSVAX® B/E, and ALVAC-HIV	Thailand	Participants in RV144clinical trial		—	[131]
		HVNT 096	NCT01799954	2012	Experimental HIV vaccine regimens using different vaccine priming combination, and boosting with the vaccines NYVAC and AIDSVAX® B/E	Switzerland	Healthy adults of both sexes		—	[132]
		HVNT 097	NCT02109354	2013	Prime: recombinant canarypox vector vaccine (ALVAC-HIV (vCP1521))Boost: AIDSVAX® B/E	South Africa	Healthy adults of both sexes		P_AE/B_/alum	[133]
		RV306	NCT01931358	2013	12, 15, or 18 months late boost since RV144 Vaccination: ALVAC-HIV + AIDSVAX® B/E, AIDSVAX® B/E, and ALVAC-HIV	Thailand	Healthy adults of both sexes		—	[134]
		HVNT 100	NCT02404311	2015	Prime: ALVAC-HIV vector (vCP2438)—expressing HIV-1 Env gp120 (subtype C), the transmembrane region of Env gp41, gag, and protease (all subtype B). Boost: Bivalent subtype C gp120/MF59®	South Africa	Healthy adults of both sexes		P_C_/MF59	[135]
		HVNT 702	NCT02968849	2016	ALVAC-HIV (vCP2438) expressing the HIV-1 envelope glycoprotein of the subtype C, along with the gp41 transmembrane sequence, gag, and protease from the subtype B LAI strain + Bivalent subtype C gp120/MF59	South Africa	Healthy adults of both sexes		Clinical trial stopped	[136]

RNA viruses	mRNA	iHIVARNA	NCT02413645	2015	Naked mRNA containing dendritic cell activation signals iHIVARNA-01 (TriMix) and encoding a novel HIV immunogen sequence (HIVACAT T-cell immunogen) (HTI))	Spain	HIV-1 infected adults of both sexes		—	[137]
		iHIVARNA phase II	NCT02888756	2017	HIVACAT-TriMix aloneTriMix alone	Belgium, Netherlands, Spain	HIV-1 infected adults of both sexes		—	[138]
		HVNT 302	NCT05217641	2022	Evaluation of the safety and immunogenicity of BG505 MD39.3, BG505 MD39.3 gp151, and BG505 MD39.3 gp151 CD4KO HIV trimer mRNA. These trimers are based on the BG505 MD39 native-like trimer reported in PMID: 27617678	US	Healthy adults of both sexes		Clinical trial not completed	—

**Table 3:** HIV-1 clinical trial that used Mosaic vaccine design.

Study ID	Clinical trials identifier	Year started	Immunogen/description	Country	Population enrolled	Note	References
IAVI B003	NCT01215149	2010	Adenovirus serotype 26 with an HIV-1 envelope A insert (Ad26.EnvA) Adenovirus serotype 35 with an HIV-1 envelope A insert (Ad35.Env)	US, Kenya Rwanda, South Africa	Healthy adults of both sexes	Homologous and heterologous regimens	[150]

MENSCH	NCT02218125	2014	MVA-vectored HIV-1 bivalent mosaic immunogen vaccine (MVA.mos1 and MVA.mos2; MVA Mosaic) which delivered two different but complementary HIV-1 *gag/pol/env* inserts	US, Kenya Rwanda, South Africa	Healthy adults of both sexes and those who had received two or three doses of NCT00618605	—	[127]

APPROACH	NCT02315703	2014	Prime: Ad26.Mos1.Env, Ad26.Mos1.Gag-Pol, and Ad26.Mos2.Gag-Pol Boost: Ad26.Mos.HIV or MVA-mosaic with or without gp140 protein	US, Rwanda, South Africa, Thailand, Uganda	Healthy adults of both sexes	Aluminum phosphate adjuvanted with clade C Env gp140 protein	[144]

CR108068	NCT02685020	2016	Ad26.Mos.HIV mosaic Env, Gag, and Pol antigens Clade C gp140	US	Healthy adults of both sexes	—	[151]

TRAVERSE	NCT02788045	2016	Prime: trivalent Ad26.Mos.HIV or tetravalent Ad26.Mos4.HIV Boost: trivalent Ad26.Mos.HIV and Clade C gp140 plus adjuvant or Ad26.Mos4.HIV and Clade C gp140 plus adjuvant	US, Rwanda	Healthy adults of both sexes	—	[152]

ASCENT	NCT02935686	2017	Prime: tetravalent Ad26.Mos4.HIV Boost: tetravalent Ad26.Mos4.HIV and Clade C gp140 plus adjuvant or a combination of Mosaic gp140 and Clade C gp140 plus adjuvant or HIV Bivalent Vaccine	US, Kenya, Rwanda	Healthy adults of both sexes	Clinical trial not completed	—

IMBOKODO	NCT03060629	2017	Prime: tetravalent Ad26.Mos4.HIV Boost: Clade C gp140 with aluminum phosphate adjuvant	Malawi, Mozambique, South Africa, Zambia, Zimbabwe	Healthy, sexually active with male partners, at risk for HIV-1, adult females	The vaccine did not prevent HIV infection in high-risk women	[146]

IGHID 11810	NCT03844386	2019	Pilot study to evaluate the safety and immunogenicity of: MVA.tHIVconsv3 and MVA.tHIVconsv4, administered alone or in combination. Expression immunogens derived from conserved yet immunogenic regions of HIV-1	US	HIV-1 infected adults of both sexes under ART	Clinical trial completed, but no publications available	—

MOSAICO	NCT03964415	2020	Prime: Ad26.Mos4.HIV is a tetravalent vaccine composed of Ad26.Mos1.Gag-Pol, Ad26.Mos2.Gag-Pol, Ad26.Mos1.Env, and Ad26.Mos2S.Env Clade C Boost: Clade C and Mosaic gp140 HIV bivalent vaccine which contains: Clade C gp140, HIV-1 Env gp140 of Clade C, Mosaic gp140, HIV-1 Env gp140, and aluminum phosphate adjuvant	Uganda	Homosexual and bisexual men and trans women	Clinical trial stopped. According to the data, those who received the vaccine continued to contract HIV at a rate that was comparable to that of the other group	—

HIV-CORE 0052	NCT04586673	2021	Safety profile evaluation of: ChAdOx1.tHIVconv1, MVA.tHIVconsv3, and MVA.tHIVconsv4	UK	Healthy adults of both sexes	Clinical trial completed, but no publications available	—

HIV-CORE 006	NCT04553016	2021	Prime: ChAdOx1.tHIVconv1 Boost: MVA.tHIVconsv3 and MVA.tHIVconsv4	Kenya, Uganda, Zambia	Healthy adults of both sexes	Clinical trial completed, but no publications available	—

## Data Availability

The data used to support the findings of this study are included within the article. Additional data are available from the corresponding author upon request.
